# Anticancer properties of bromelain: State-of-the-art and recent trends

**DOI:** 10.3389/fonc.2022.1068778

**Published:** 2023-01-09

**Authors:** Raffaele Pezzani, Manuel Jiménez-Garcia, Xavier Capó, Eda Sönmez Gürer, Farukh Sharopov, Tchokouaha Yamthe Lauve Rachel, David Ntieche Woutouoba, Antonio Rescigno, Stefania Peddio, Paolo Zucca, Patrick Valere Tsouh Fokou, Miquel Martorell, Zehra Gulsunoglu-Konuskan, Alibek Ydyrys, Tynybekov Bekzat, Tussupbekova Gulmira, Christophe Hano, Javad Sharifi-Rad, Daniela Calina

**Affiliations:** ^1^ Phytotherapy Lab, Endocrinology Unit, Department of Medicine (DIMED), University of Padova, Padova, Italy; ^2^ Associazione Italiana per la Ricerca Oncologica di Base (AIROB), Padova, Italy; ^3^ Laboratory of Neurophysiology, Biology Department, University of Balearic Islands (UIB), Palma de Mallorca, Spain; ^4^ Research Group in Community Nutrition and Oxidative Stress and Health Research Institute of the Balearic Islands (IdISBa), University of Balearic Islands, Palma de Mallorca, Spain; ^5^ Faculty of Pharmacy, Department of Pharmacognosy, Sivas Cumhuriyet University, Sivas, Turkey; ^6^ Research Institution “Chinese-Tajik Innovation Center for Natural Products” of the National Academy of Sciences of Tajikistan, Dushanbe, Tajikistan; ^7^ Institute of Medical Research and Medicinal Plants Studies (IMPM), Yaounde, Cameroon; ^8^ Antimicrobial and Biocontrol Agents Unit, Department of Biochemistry, Faculty of Science, University of Yaounde, Yaounde, Cameroon; ^9^ Department of Biomedical Sciences, University of Cagliari, Cagliari, Italy; ^10^ Department of Biochemistry, Faculty of Science, University of Bamenda, Bambili, Cameroon; ^11^ Department of Nutrition and Dietetics, Faculty of Pharmacy, and Centre for Healthy Living, University of Concepción, Concepción, Chile; ^12^ Universidad de Concepción, Unidad de Desarrollo Tecnológico, UDT, Concepción, Chile; ^13^ Faculty of Health Science, Nutrition and Dietetics Department, Istanbul Aydin University, Istanbul, Turkey; ^14^ Biomedical Research Centre, Al-Farabi Kazakh National University, Almaty, Kazakhstan; ^15^ The Elliott School of International Affairs, George Washington University, Washington, DC, United States; ^16^ Department of Biodiversity and Bioresources, Al-Farabi Kazakh National University, Almaty, Kazakhstan; ^17^ Department of Biophysics, Biomedicine and Neuroscience, Al-Farabi Kazakh National University, Almaty, Kazakhstan; ^18^ Department of Biological Chemistry, University of Orleans, Chartres, France; ^19^ Facultad de Medicina, Universidad del Azuay, Cuenca, Ecuador; ^20^ Department of Clinical Pharmacy, University of Medicine and Pharmacy of Craiova, Craiova, Romania

**Keywords:** bromelain, cancer, chemotherapeutics, cytotoxicity, apoptosis, molecular targets

## Abstract

Bromelain is a key enzyme found in pineapple (*Ananas comosus (L.) Merr*.); a proteolytic substance with multiple beneficial effects for human health such as anti-inflammatory, immunomodulatory, antioxidant and anticarcinogenic, traditionally used in many countries for its potential therapeutic value. The aim of this updated and comprehensive review focuses on the potential anticancer benefits of bromelain, analyzing the cytotoxic, apoptotic, necrotic, autophagic, immunomodulating, and anti-inflammatory effects in cancer cells and animal models. Detailed information about Bromelain and its anticancer effects at the cellular, molecular and signaling levels were collected from online databases such as PubMed/MedLine, TRIP database, GeenMedical, Scopus, Web of Science and Google Scholar. The results of the analyzed studies showed that Bromelain possesses corroborated pharmacological activities, such as anticancer, anti-edema, anti-inflammatory, anti-microbial, anti-coagulant, anti-osteoarthritis, anti-trauma pain, anti-diarrhea, wound repair. Nonetheless, bromelain clinical studies are scarce and still more research is needed to validate the scientific value of this enzyme in human cancer diseases.

## 1 Introduction

Cancer has become a global problem, with a negative impact on patients, their families and the whole of society. Tumor cells can affect any part of the body and are characterized by proliferation caused by the loss of control of cell division, reducing the possibility of repairing cell DNA mutations, the ability to avoid genetically programmed cell death (apoptosis), to form new blood vessels (angiogenesis), to invade other tissues and develop metastases, the ability to hide from the action of the immune system or the loss of abilities to fulfil a function in the body (1, 2). The main objectives of oncological treatments are the remission or cure of the disease, the extension of the duration and the improvement of the patient’s quality of life. The effectiveness of oncological therapies depends on early detection, accurate diagnosis of cancer, compliance with standards of care and the correct way of administering treatments. Chemotherapy is a systemic treatment, administered to destroy tumor cells or stop their multiplication (3). The cytotoxic action also manifests itself in healthy cells that multiply rapidly (eg hair, and cells of the intestinal mucosa), causing adverse reactions (4). Chemotherapy is administered strictly according to the type of tumor cell and the patient’s response to treatment (5, 6). Today, there are a variety of natural cancer treatments that may be able to provide results comparable to chemotherapy and radiation therapy (7, 8). They are much less toxic and invasive compared to standard methods of cytostatic treatment. Some of the most successful cancer treatment plans involve a combination of therapies (9–11).

**Figure 1:**
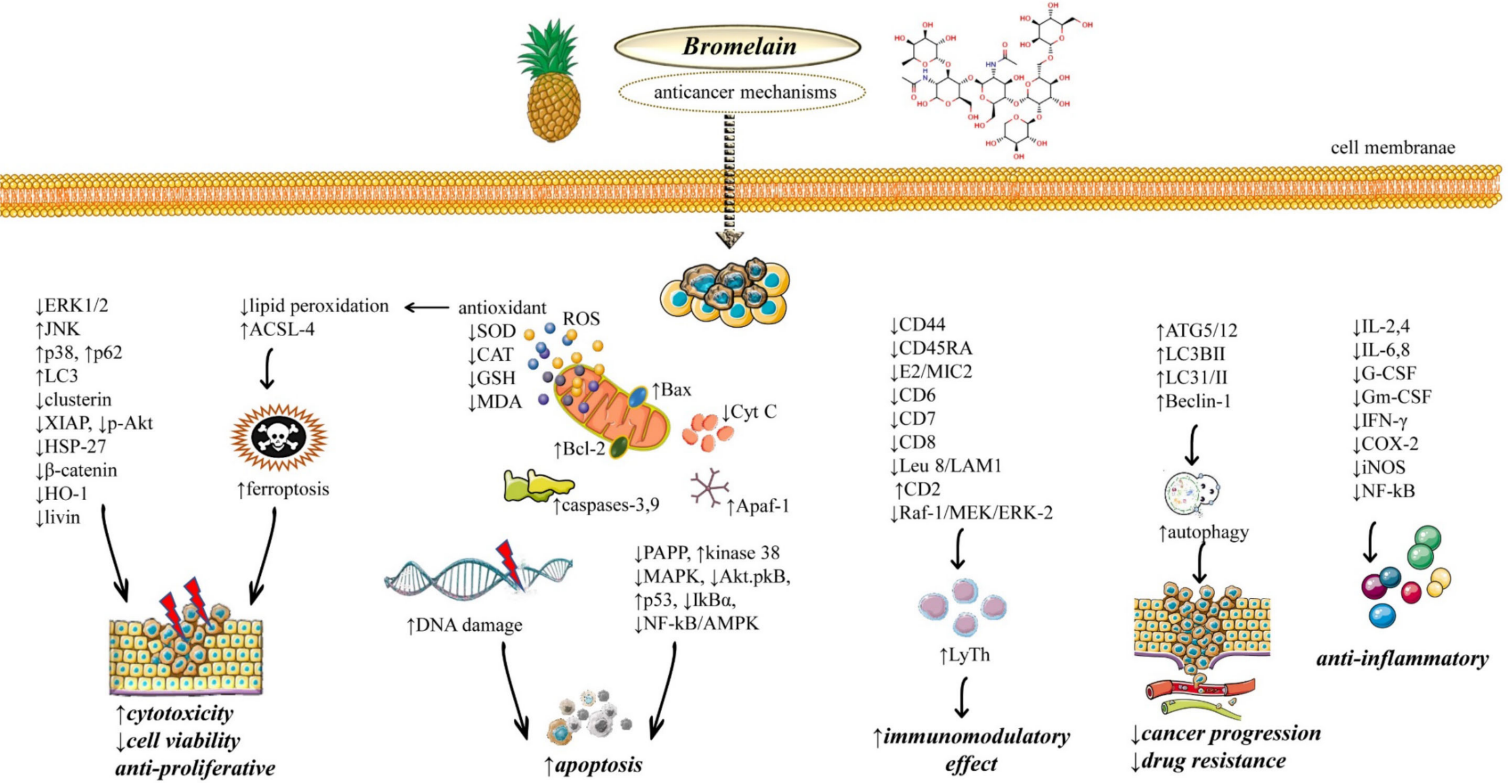
Illustrative diagram with the most representative anticancer molecular mechanisms of Bromelain. Abbreviations and symbols: ↑increase, ↓decrease, extracellular signal-related kinase (ERK), c-Jun N-terminal kinase (JNK), X-linked inhibitor of apoptosis protein (XIAP), Heat shock protein 27 (HSP27), Heme oxygenase-1 (HO-1), long-chain fatty acyl-CoA synthetase 4 (ACSL4), reactive oxygen species (ROS), superoxide dismutase.(SOD), Glutathione (L-γ-glutamyl-L-cysteinyl-glycine; GSH), malondialdehyde (MDA), Bcl-2 Associated X (Bax), B-cell lymphoma 2 (Bcl-2), Pro-Apoptotic Apoptosis Protease–Activating Factor 1 (Apaf-1), mitogen-activated protein kinase (MAPK), serine/threonine protein kinase (Akt), nuclear factor-kappa B (NF-κB), cluster of differentiation 44 (CD44), serine/threonine kinase (Raf1), mitogen-activated protein kinase (MEK), Autophagy related 5 protein (ATG5), Microtubule-associated protein 1A/1B-light chain 3 (LC3), IL (interleukin), Interferon-gamma (IFNγ), cyclooxygenase-2 (COX-2), Granulocyte-macrophage colony-stimulating factor (GM-CSF), granulocyte-colony stimulating factor (G-CSF).

Bromelain is an enzyme with a particular proteolytic activity that can be easily obtained from the pineapple stem (*Ananas comosus* (L.) Merr.). Bromelain is also present in other parts of the pineapple and is found in association with other cysteine proteases (12). Bromelain is traditionally extracted from the juice following ultrafiltration and centrifugation, with an end process culminating in lyophilization (13). However, the growing request for this substance induced the research to develop new purification techniques, i.e. ion exchange chromatography, gel filtration, aqueous two-phase extraction, affinity chromatography, reverse micelle chromatography, and recombinant DNA technology, with the final aim to contain costs and increase the production yield (14). Given the elevated costs of production and a market that needs a highly purified and certified bromelain, pineapple cultivation is widespread in many tropical and subtropical areas, i.e. South-East Asia, Africa, and Central-South America (15). At the basis of this great request for bromelain, there are the extraordinary properties that make it indispensable for numerous processes related to foods production, cosmetics, pharmaceuticals, and textile industries (13, 15–18). In addition, since its discovery, bromelain demonstrated interesting pharmacological and potential medical applications. In 1875, bromelain was first analyzed as a specific substance obtained by pineapple (19) and later in 1892, other researchers defined their matter of investigation as “bromelin” (20). After in 1962, Seligman and collaborators demonstrated the anti-inflammatory effect of bromelain (21). Since this finding, bromelain has been increasingly studied in different settings, leading to the discovery of numerous properties in human health, i.e. treatment of osteoporosis and osteoarthritis, diarrhea, treatment of chronic wounds, surgical debridement, anti-edema and anti-inflammatory processes, trauma pain, burn eschars, and many others (**Figure 1**) (22–29). Certainly, such clinical studies have been based on previous animal or cellular experiments. Indeed, a plethora of preclinical works have been published on this substance as they will be reported in this review. In particular, this updated review is focused on the anti-cancer effect of bromelain, an activity that emerged since 1972, when a first work explored the use of the enzyme in malignant tumors with notable remissions in some anecdotal cases (30).

## 2 Review methodology

To carry out this updated review on the anticancer effects of bromelain, published studies were searched in specialized databases such as PubMed/MedLine, TRIP database, GeenMedical, Scopus, Web of Science and Google Scholar, using the next MeSH terms: ‘Animals”, “Anticarcinogenic Agents/pharmacology”, “Antineoplastic Agents/pharmacology”, “Bromelain/pharmacology”, “Bromelain/administration and dosage”, “Bromelain/chemistry”, “Cell Line, Tumor”, “Cell Survival/drug effects”, “Dose-Response Relationship”, “Drug, Humans”, “Nanocapsules/administration and dosage”, “Neoplasms/drug therapy”, “Neoplasms, Experimental/pathology”, “Signal Transduction/drug effects”, “Treatment Outcome”. Each article published before 1 August 2022, written in English, and their significance was evaluated by analyzing the title and abstract. All studies written in preclinical and clinical language that highlighted the anticancer molecular mechanisms, signaling pathways and action targets of bromelain were included. Duplicate studies, abstracts, presentations at conferences or that had associated homeopathic preparations were excluded. The chemical forms were validated with Chemspider and the scientific names of the plant species were validated according to WorldFloraOnline (31, 32).

## 3 Chemistry data of bromelain

### 3.1 Physicochemical characteristics

The term “bromelain” initially referred to a mixture of proteases produced by any member of the *bromeliaceae* family (14). However, the extraction is most common from pineapple fruit (*Ananas comosus*). These extracts usually contain several enzymes (including proteases, phosphatases, nucleases, oxidoreductases, and glycosidases), but the most characteristics among them are some cysteine endopeptidases that specifically hydrolytically cleave non-terminal peptide bonds (33, 34). Four main enzymes have been identified, as shown in **Table 1**.

**Table 1:** summary of the most prominent characteristics of pineapple cysteine proteases (adapted from (Colletti et al., 2021; Murachi, 1976; Rowan & Buttle, 1994) (33).

Protease	M_r_ (kDa)	pI	Optimum pH	Glycoprotein
Bromelain (fruit)	25-31	4.6	3-8	Yes
Bromelain (stem)	23.8-27	9.5	6-7	Yes
Ananain	23.4-25	>10	~7	No
Comosain	24.5	>10	~7	Yes

Proteolytic activity is present in almost all parts of the plants, but the stem and fruit are indeed richer. The main enzyme is bromelain. This is α + β protein that can be extracted both from the stem (EC: 3.4.22.32) and from fruit (EC: 3.4.22.33). The first is the most abundant showing MW of about 23.8 kDa, a pI of 9.5 and a pH optimum around neutrality (33). Bromelain from the fruit on the contrary has a slightly higher MW (25 kDa), a more acidic pI (4.6) and a broader pH optimum (3-8). All these differences on the whole may be, at least in part, responsible for the very different results observed in all the clinical and preclinical studies (33, 35). Stem bromelain is a single polypeptide chain (211/212 aminoacids), presenting at least a single glycosylation point accountable for the 23.8 kDa MW. A crucial glutamate residue (Glu68) has been suggested responsible for the preference for positively charged substrates (36). Both enzymes could undergo however complex glycosylation patterns, increasing the global MW. These reactions depend on a lot of factors and are still largely poorly known (34). The aminoacidic sequence of stem bromelain is quite homologous to other plant cysteine-peptidases such as papain, chymopapain and actinidin (37). Nonetheless, several authors reported that this proteolytic activity could be only marginally connected to the pharmacological effects. Other components (protein and non-protein) could be responsible for a complex network of interactions and synergistic actions resulting in pharmacological effects (16). Stem bromelain is very stable when frozen at -20°C, whereas a complete loss activity occurs when boiled at 100°C for 10’ (38). Optimum activity has been found at 30°C and neutrality for the glycosylated form. Both heating (40-60°C) and acidification (pH 3-4) led to significant inactivation (39). The deglycosylated form was found to be more susceptible to temperature and organic solvent inactivation, suggesting an important role of the oligosaccharide portion in the retention of the enzyme’s native form (39).

Fruit bromelain showed a similar pH optimum around neutrality and a loss of activity for a pH higher than 8.0 (40). The enzyme retained a part of catalytic activity (respectively 22% and 44%) after 30 days of storage at 30°C and 60 days of storage at 4°C (41). On the contrary, 180 days of freezing at -4°C led to an almost complete retaining (75%) of proteolytic power. Slightly different results were obtained by Chaurasiya and colleagues (40). In this study after 9 days at 5-8°C the loss of activity was significant (around 30% residual activity) (40). These differences could be explained by the different degrees of purification. Glutaraldehyde crosslinking has been shown to increase thermal and pH stability, up to 60°C and pH 8 (42). Even the effect of different organic solvents led to less marked losses of proteolytic activity. Recently, Zhou and coworkers showed that thermal inactivation followed the first-order reaction, and that half-life from 45 to 75°C decreased from 81’ to 5’ (43).

### 3.2 Purification of bromelain

Protein purification is always a challenging task since all proteins show similar physicochemical properties (being constituted always by the same 20 aminoacidic building blocks). In addition, all the required purification techniques must be performed under very controlled conditions to avoid protein inactivation (and consequently the complete loss of commercial value of the protein). Temperature, pH, presence of organic solvents, ionic strength, dilution or concentration, and various chemical and biological contaminants are all factors that should be carefully monitored and controlled during purification, affecting the overall cost of the process. In accordance, a lot of attention has been paid to the purification strategies, looking for the most effective approaches, also from an economical point of view. Up to now the most used protocol encompass the preparation of a crude bromelain extract, using conventional low-resolution techniques (44). After cleaning, peeling and cutting down the whole pineapple, cell lysis is usually obtained mechanically without any further water addition. This homogenization, after centrifugation, allows for recovering an active extract presenting however several impurities. The product can be then concentrated to improve the purification fold. Size exclusion membranes (cut-off > 5-10kDa) are usually the basis for ultrafiltration techniques, giving encouraging results (45–47). Such a strategy is quite simple and the costs are relatively low. A more expensive way to concentrate protein is lyophilization (freeze-dry). The transition of water from solid to gas form under a deep vacuum requires a high amount of energy but allows a complete solvent removal (not possible with membrane filtration). On the negative side, the stability of bromelain during freeze-drying is not high (35, 48) possibly suggesting the use of a stabilizer. All these approaches lead to a crude extract in which bromelain has been isolated from non-protein contaminants, but no real protein purification has been done. This can be achieved with high-resolution techniques, that greatly increase the final cost of the purified product. Usually low- and high-resolution isolation steps are combined following the purpose of the purified extract (Arshad et al., 2014). One of the easiest ways to fractionate proteins is reversible precipitation. Several approaches can be used, including salts, isoelectric, organic solvents and non-ionic polymer precipitation. All these techniques have been tested for bromelain, with various results (40). Ethanol precipitation gave better yields than isoelectric, PEG and ammonium sulfate (48, 49). PEG can be also used in aqueous two-phase systems, preserving bromelain activity (50, 51). Precipitation is a very affordable option, with quite good recovery yields. However, the obtained purification fold factors are not very high, and the product could be contaminated by salts or polymers (33).

To improve protein purification fold, the golden standard in biochemistry is chromatography. This term encompassed all the techniques using a combination of two immiscible phases, moving one on the other. The analytes are separated giving their different affinity for the two phases. Chromatographic separation typically highly increases the protein purification yields, but with higher operational costs. Often chromatography is employed in combination with fractionated precipitation (52). Ion exchange chromatography relies on reversible electrostatic interaction between a resin and charged analytes. Such an approach has been proved able to remove contaminant polysaccharides (35), increasing bromelain-specific activity (even more than salt precipitation) (53, 54). Ion exchange has been also tested in combination with size exclusion chromatography, resulting in a 41% recovery of initial activity (55).

Affinity chromatography (AC) is a very potent separation technique, relying on a specific interaction between a protein and a ligand-functionalized chromatographic column (56). This could allow (theoretically) to completely purify proteins in a single step. Bobb and colleagues achieved a similar result using an e-aminocaproic-d-tryptophan methyl ester coupled with Sepharose 4B (57). In this case stem, bromelain was effectively purified to a single SDS-PAGE band in one step. Alternatively, Rowan et al. used acetone precipitation and a Sepharose- Ahx-Phe-GlySc resin to purify fruit bromelain (Rowan et al., 1990). In both cases, mercury was used to allow elution after sample application and resin washing of unbound. The downside of such approaches is the high costs of similarly functionalized resins. In the case of recombinant bromelain, His-tag could be inserted allowing AC purification based on nickel/nitriloacetic aced-based resins (58, 59). High recovery yields of bromelain have been also obtained using reversed micellar extraction (RME) process (40, 60, 61). RME relies on the inclusion of proteins into micellar inner cores, and the subsequent release in the aqueous phase. The works reported good bromelain recovery and significant enhancement in the purification fold.

### 3.3 Determination of bromelain activity

Casein and azocasein are the most used substrates for the spectrophotometric determination of bromelain activity. Casein is chosen due to its low cost and high availability (62). The reaction is performed in 0.5-1% casein solutions at neutral pH. After 5-30’ at 37°C trichloroacetic is added to stop the reaction and the resulting soluble peptides are spectrophotometrically detected at 280 nm, comparing absorbance with a tyrosine standard curve (48, 63). Reducing thiols and EDTA is required to, respectively, prevent oxidation of cysteine in the active site and chelate interfering metals (64, 65). Upon proteolysis, azocasein releases instead diazo sulphonic analogues of Tyr and His residues, quantifiable at 440 nm. Operational conditions are like casein, but alkali addition is necessary to develop color (66). Also, in this case, the reaction is performed at 37°C. As alternatives, several synthetic substrates are available usually based on chromogenic or fluorogenic moieties (such as *p*-nitrophenol or *p*-nitroaniline) coupled to small peptides. For instance, Z-Arg-Arg-p-nitroaniline, Bz-Phe-Val-Arg-pNA, *p*Glu-Phe-Leu-*p*-nitroanilide and N-Cbz-L-Gln-*p*-nitrophenyl ester hydrolysis leads in solution to chromophores easy detectable at 405 nm (67, 68). These approaches are easy, convenient and quick. One more advantage is the fact that different substrates present different specificity for various bromelain forms. For instance, Z-Arg-Arg is more specific for stem enzymes (69, 70). On the other hand, fruit bromelain showed a preference for Bz-Phe-Val-Arg.

## 4 Bromelain as an emerging anticancer agent: Molecular mechanisms, signaling pathways and therapeutic targets

To elucidate in detail the potential mechanisms of anticancer action, bromelain has been extensively studied in numerous studies (16, 71–73). Bromelain anticancer effects were evaluated in both “*in vivo”* in animal models and “*in vitro”* as a potential agent against several cancer lines such as gastrointestinal, colorectal, breast, mesothelioma, lung, hepatic, pancreatic, epidermoid, and melanoma (71) (**Table 2** and **Figure 1**).

**Table 2:** Table with summarized anticancer mechanisms of Bromelain from *in vitro* studies.

Type of tested cancer cell lines	Effects	Results/Potential Mechanisms	Ref
MDA-MB231breast adenocarcinoma A2058 melanoma OCI-LY19 non-adherent human B cell lymphoma HL-60 human leukemia cells	↑cytotoxicity anti-proliferative	IC_50 =_ 0.3 µM ↑ cancer cells death in MDA-MB231, A2058 IC_50 =_ 1 µM ↑ cancer cells death in OCI-LY19, HL-60	(36)
MCF-7, MDA-MB-231 breast adenocarcinoma	↑cytotoxicity ↑autophagy ↑apoptosis	IC_50 =_ 1 µg/mL ↓ cancer cells viability: 20% for MCF-7, 30% for MDA-MB-231 IC_50 =_ 100 µg/mL ↓cancer cell viability, ↓caspase 9, ↓ERK½, ↑JNK, ↑p38 60% for MCF-7, 80% for MDA-MB-231 ↑autophagic markers: ↑LC3BII, ↑beclin-1	(74)
MCF-7 breast adenocarcinoma	↓ proliferation	nanocapsules of bromelain ↓cells proliferation 72.6% at IC_50 =_ 1.25 µg/mL 66.4% at IC_50 =_ 0.625 µg/mL	(75)
	↓cells viability	↑cytotoxicity IC_50 =_ >75 µg/mL	(76)
	↓cells viability	anti-proliferative 80% similar to taxol at IC_50_ > 50 µg/mL	(77)
4T1 breast adenocarcinoma	↓tumor mass ↑cytotoxicity	combination treatment of bromelain + cisplatin reduced tumor cells growth ↓GREM1 ↓IL-1β, ↓IL-4, ↓NF-kB, ↓PTGS2, ↓NO	(78)
MDA-MB-231 breast adenocarcinoma	↓cells viability ↑apoptosis	combination treatment bromelain IC_50 =_ 2 µM + cisplatin IC_50 =_ 1.5 µM ↓ cell viability to 50% bromelain IC_50 =_ 0.9 µM + cisplatin IC_50 =_ 4.0 µM ↓ cell viability to 70% ↓anti-apoptotic proteins: ↓cIAP 1, ↓Bcl-2, ↓CAT, ↓clusterin, ↓HO-1, ↓livin, ↓XIAP, ↓claspin, ↓HSP27 ↓IkB kinase, ↓NF-kB no effect on pro-apoptotic protein Bax	(79)
GI-101A breast adenocarcinoma	↓cells viability ↑apoptosis ↑cytotoxicity	IC_50 =_ 5, 10, 20, 40, 50 µg/mL ↓cell viability 36% ↑apoptosis ↑DNA fragmentation ↑JNK, ↑kinase 38 ↑caspase-3, ↑caspase-9	(80)
MKN45, KATO-III, LS174 gastrointestinal carcinoma	↓cells survival ↑autophagy ↑apoptosis ↑mucin depletion	↓cells survival IC_50 =_ 100 -600 µg/mL for MKN45, KATO-III IC_50 =_ 30 µg/mL for LS174 synergic anticancer effects with N-acetylcysteine ↑caspase-3, 7, 8, ↓PARP, ↓cytochrome C ↓LC3-II, ↓Bcl-2, ↓ p-Akt, ↓Atg3, ↓Atg5, ↓Atg7, ↓Atg12, ↓Beclin 1, ↓MUC2, ↓MUC5AC	(81, 82)
AGS gastric carcinoma	↑cytotoxicity anti-proliferative	↑cytotoxicity IC_50_ >50 µg/mL	(76)
MPM malignant peritoneal mesothelioma	↓cells viability ↑autophagy ↑apoptosis	↓cell viability 60% at IC_50 =_ 100 µg/mL ↑caspase-3, 7, 8, 9, ↑Cyt C ↓PARP, ↓ p-Ikkb, ↓NF-κB,↓ p-Akt	(83, 84)
SCC25 oral squamous carcinoma	↓cells viability	↓cell viability 31.07% at IC_50 =_ 25 μg/mL	(85)
HepG2 hepatocellular carcinoma	↓cells survival ↑apoptosis ↓ cells proliferation ↓cells growth ↑ cell cycle arrest in G2/M phase	↓cell viability IC_50 =_ 25 μg/mL antioxidant in cell cancers: ↓SOD, ↓CAT, ↓GSH, ↓MDA ↑apoptosis: ↑p53 ↓β- catenin	(86)
PC3 prostate cancer	↓cell survival ↑apoptosis	↓cell viability 25% at IC_50_ >50 µg/mL	(76)
A431 epidermoid carcinoma A375 melanoma	↓cell survival ↑apoptosis	↑cytotoxicity IC_50 =_ 200mg/mL for A431 IC_50 =_ 400mg/mL for A375 cells ↑Bax, ↑Apaf-1, ↑caspases-3, 9, ↓Bcl-2	(87)
DLD-1, HT-29, HCT116 colorectal carcinoma	↑cytotoxicity ↑autophagy	IC_50_ = 50 μg/ml for HCT116 IC_50 =_ 70 μg/ml for HT-29, DLD-1 ↑AIF, ↑Endo G, ↑caspases -3, -8, -9, ↑PARP-1, ↑ATG5/12, ↑Beclin, ↑p62, ↑LC3	(88)
Non-*Kras* mutant (Caco2 and NCI-H508) *Kras* mutant (HCT-116, DLD-1) colorectal carcinoma	↓cell viability ↑ferroptosis	↑cytotoxicity IC_50 =_ 50 µg/mL Kras mutant cells were more susceptible ↓ACSL-4, ↑ miRNAs in Caco2 cells ↑ROS, ↑feroptosis *Kras* mutant cells	(89)
DMBA-TPA-induced mouse skin cancer cells	↑apoptosis ↓papilloma cells development	↓tumorigenesis ↓IκBα, ↓NF-κB, ↓COX-2, ↓Bcl-2, ↑Bax, ↑caspase 3, 9, ↑p53, ↓ERK1/2, ↓MAPK, ↓Akt	(90)
K562 human acute myeloid leukemia	↑apoptosis	↑peroxidase, ↑ROS, ↑Bax, ↑caspase-3, ↑Cyt C, ↓Bcl2, ↑p53	(91)
DLA Ascitic Dalton’s lymphoma cells from mice	↑apoptosis	↑ROS, ↓Cyt C, ↓Bad, ↓Bax protein ↑NF-kB, ↑Bcl-2, x↑serum vitamin C, E	(92)
Mouse lung adenocarcinoma cells	↓carcinogenesis ↓inflammation ↓oxidative stress	bromelain plus ethanolic olive leaf extract ↑Nrf2, ↓NF-κB translocation, ↓IL-6, ↓TNF-α, ↓MMP2, ↓MMP9, ↑LPO, ↑ROS	(93)
TFK-1, SZ-1, cholangiocarcinoma cells	↑cytotoxicity ↑apoptosis	↓cells viability IC_50 =_ 150 μM ↓NF-κB/AMPK, ↓p-AKT, ↓p-ERK, ↓p-STAT3, ↓MMP9 ↑PARP, ↑p-AMPK	(94)
C57BL/6N strain mice musculocutaneous flaps	↓necrotic inflammation	↓tissue necrosis by 25% ↓apoptotic cells, ↑angiogenesis	(95)

**↑**increase, ↓decrease, tumor inflammatory genes Gremlin (GREM1), interleukin 1β (IL-1β), interleukin-4 [IL-4], nuclear factor κB subunit 1 (NFκB1), and prostaglandin-endoperoxide synthase 2 (PTGS2), extracellular signal-regulated kinase ½ (ERK½), c-jun N-terminal kinase (JNK), light chain 3 protein B II (LC3BII), c-Jun N-terminal kinase (JNK), poly(ADP ribose) polymerase (PARP)-1, nuclear factor-kappa B (NF-kappaB), cyclooxygenase-2 (Cox-2), extracellular signal-regulated protein kinase (ERK1/2), mitogen-activated protein kinase (MAPK), 7,12-dimethylbenz(a)anthracene (DMBA)-initiated and 12-O-tetradecanoylphorbol-13-acetate (TPA), metalloproteinases MMP.

### 4.1 Cytotoxicity

The cytotoxic properties of bromelain have been known since ancient times, as it was used in traditional Asian medicine. Cytotoxic or anticancer bromelain effects are associated with its protease activity (16). In SCC25 human oral squamous carcinoma cells, cell viability decreased in a dose‐ and time‐dependent manner after 24‐hr treatment with bromelain, from 95.16% (at 0.78 μg/mL) to 69.93% (at 25 μg/mL) (85). It has been reported that bromelain-induced cytotoxic activity in human gastrointestinal cancer cells (76, 96), breast cancer cells (76, 80), and hepatocellular carcinoma cell lines (97). It is also evidenced that bromelain presented antiproliferative properties in gastric and colon carcinoma cells. For example in human gastrointestinal cell lines, in the case of MKN45 cells, the enzyme in a range of concentration from 100 to 600 µg/mL but non-dose dependently reduced cell survival at 72h of treatment (81). In the case of KATO-III cells, antiproliferative effects were shown at concentrations above 100 µg/mL, while in LS174 cells, cytotoxic properties of bromelain were evidenced above 30 µg/mL and its cytotoxicity increased with higher bromelain concentrations at 72h (81). Cytotoxicity of bromelain was also evaluated in other human gastric carcinoma cells (AGS cells) at concentrations from 5 to 600 µg/mL, whereas the first anti-proliferative effects (25% growth reduction) were observed at concentrations >50 µg/mL in a dose-dependent manner at 24h of treatment (76). Another study demonstrated a reduction of almost 75% cell viability after 12 hours of treatment with a concentration of 50 µg/mL bromelain in DLD-1 colorectal carcinoma cells (89).

Cytotoxic properties of bromelain were also tested in hepatocellular carcinoma cells (HepG2). Cells treated for 48 hours with bromelain at 25 µg/mL, 50 µg/mL, 100 µg/mL, and 125 µg/mL, showed a decrease in cell viability in a concentration-dependent manner (86). Bromelain cytotoxic effects were also tested in malignant Peritoneal Mesothelioma (MPM cells). This study showed a reduction of 60% in cell viability after 4 hours of treatment with 100 µg/mL, while at 72 hours of treatment the reduction in cell viability was much higher (>80%) (83).

Human breast cancer cells were frequently used to study the cytotoxic effects of different bioactive compounds (98–100). Azarcan and their collaborators showed that bromelain at 0.3 µM induced high mortality in breast adenocarcinoma and melanoma cell lines (36). Bhui and colleagues showed that bromelain in a dose and time-dependent manner induced a cytotoxic response in MCF-7 cells after 72 or 96 hours of treatment (74). Bromelain induced a reduction in cell proliferation of 72.6% (at 1.25 µg/mL) and 66.5% (at 0.625 µg/mL) at 24h in MCF-7 cells, however in this case bromelain was mobilized in nanocapsules surface by coordination with Zn^+2^ facilitating the entry into the cell (75). Similarly to previous data, Raeisi and collaborators tested the effects of bromelain in MCF-7 cells, where concentrations >75 µg/mL was able to induce cytotoxicity greater than 70% at 24h of treatment (76). Again in MCF-7 cells, another work established that bromelain at 50 µg/mL for 48h could reduce by 80% cell viability in a similar way that taxol (77). In GI-101A cells human breast cancer line, a decrease in viable cell number after 24h of treatment with different concentrations of bromelain (5, 10, 20, 40, and 50 µg/mL) was demonstrated (80). In particular, at 20 µg/mL, bromelain diminished the percentage of viable cells to 36%, while higher doses caused cell death in the range of 95% or greater (80). Cytotoxic effects were also reported in another human cell line (PC3 prostate cancer cells) where a 25% reduction of cell viability was observed at concentrations >50 µg/mL in a dose-dependent manner (Raeisi et al., 2019).

### 4.2 Apoptosis

Apoptosis is a basic cell death mechanism, is vital in physiological processes, maintaining cellular homeostasis, and is detrimental in pathological processes, for example in tumour suppression (98, 101–104). This cell death process can be responsible for blocking cancer cell spread, intervening in the inhibition of the metastasis process (105, 106). Apoptosis has two pathways to be carried out, one that is considered internal, in which various families of proteins are implicated, and a second pathway, in this case external, in which different ligands bind to specific receptors (107). In both cases, various caspases, key proteins in executing the apoptotic signals, will be activated (108–110). Bromelain has been extensively studied for its implication in cell death, indeed it can intervene in the expression of Bcl-2, a type of protein with an anti-apoptotic effect but can also positively regulates the expression of apoptotic genes, such as Bax, Apaf-1, and caspases-9 and -3 (87). *In vitro* studies have shown that bromelain can induce apoptosis. In breast cancer cells, specifically in GI-101A cells, the use of this proteolytic enzyme positively regulates the N-terminal kinase c-Jun and kinase 38, in addition to improving the activity of caspases -3 and -9 at 24h (80). Moreover, bromelain’s capacity to increase apoptosis has been shown in other cancer types. In different lines of colorectal cancer, the levels of apoptosis-inducing factor (AIF), Endo G, and caspases -3, -8, and -9 increased once the cells have been treated with bromelain (88). In another study with colorectal cancer cell lines, bromelain stimulated a process called ferroptosis (89).

Ferroptosis is a type of cell death, also programmed, which depends on iron, and is characterized by an accumulation of lipid peroxides. Currently, this type of cell death is being studied as a mechanism to combat cancer cells (111) (112). In this study, an increase in the expression of ACSL-4 has been observed after treatment with bromelain in Caco2 cells (89). This protein is responsible for ferroptosis to take place, thus bromelain also exerted cytotoxic effects against colorectal cancer cells and could reduce polyp number and submucosal layer length in mice (89). From another study, it has been possible to corroborate the effect of bromelain with other enzymes or pathways that intervene in apoptotic processes. This is the case of cyclooxygenase-2 (Cox-2) or the MAPK and Akt/protein kinase B (PKB) pathways, all of which downregulate their expression (90). In particular, bromelain acted by inhibiting the phosphorylation of IκBα, which triggers the retention of NF-κB in the cytosol, decreasing the expression level of Cox-2 (90).

In human cholangiocarcinoma cells (TFK-1, SZ-1), bromelain (and papain) blocked NF-κB/AMPK signalling, inducing cytotoxic effects through the activation of apoptosis in both cell lines after 48h of treatment (94). Apart from apoptosis, the anti-cancer effects of bromelain appear to be also controlled by necrosis, a process in which cell death occurs because of adverse external cell conditions such as injury, trauma, or infections that lead to unregulated digestion of cell components (113). In a recent study with C57BL/6N strain mice, the use of bromelain reduced tissue necrosis by 25% (95). A higher density of functional microvessels was lost compared to controls, as well as a decrease in myeloperoxidase-positive neutrophils, and a reduction in apoptotic cells (95). Therefore, the use of bromelain could be considered in the future as a therapy to prevent cell necrosis, a harmful cell process that often results in inflammation and tissue damage (95).

### 4.3 Autophagy

Another process in which the implication of bromelain has been verified is autophagy. This mechanism focuses on the degradation and recycling of cellular components (107, 114, 115). In the case of cancer, this process has a dual activity, since it is capable of promoting the survival of cancer cells, through protective autophagy, or participating in the death of these cells, inducing cytotoxic autophagy (116, 117). In normal tissues, autophagy at the basal level is necessary as it provides housekeeping functions as well as increased apoptosis (118, 119). Even in periods of stress and hunger, the autophagy process is necessary (120). Differently, in cancer cells this process can allow them to survive metabolic stress, for example, being a source of energy in the face of a possible lack of nutrients, which is why its inhibition or activation in cancer cells is not completely understood (120). Although autophagy may present this duality, various studies showed that bromelain participated in the reduction of cells of different cancer types through the activation of this process. In MCF-7 cells treatment with bromelain increased the autophagic process and slow down their growth (74). In that same study, pre-treated cells with 3-methyladenine (autophagy inhibitor) demonstrated a decrease in the apoptotic process, thus showing that autophagy can affect the onset of apoptosis (74). In colorectal cancer cells, it has been shown an increased level (2 to 3 times) of autophagy with the creation of lysosomes in the cells treated with bromelain. Concurrently, levels of proteins related to autophagy were enhanced, such as ATG5/12, Beclin-1, p62, and LC3I/II (88).

### 4.4 Immunomodulatory

The immunomodulatory effects of bromelain have been studied extensively, showing that it can activate or suppress the mammalian immune system (121). One of the first researches was conducted on human T cells from bromelain-treated PBMC stimulated by mitogenic CD2 mAb to explore the migration and activation of lymphocytes (122). Bromelain acted by removing the extracellular domains of the CD44, CD45RA, E2/MIC2, CD6, CD7, CD8, and Leu 8/LAM1 surface molecules and thus enhancing T cell activation through CD2. The continuation of the research expanded the use of bromelain in monocytes and granulocytes and extended the surface molecules affected by the enzyme, again remarking on the promoting effects on adhesion and activation of immune cells (123). Also, human peripheral blood lymphocytes were pretreated with bromelain to study the anti-proliferative activity in different cancer cell lines (124). The authors reported that lymphocytes were boosted by the enzyme and could enhance the immune system response to the fight against cancer at least *in vitro* experiments. More strictly to human studies, in 16 breast cancer patients treated for 10 days, bromelain could reduce CD44 expression (similar to above), while augmenting CD11a and CD62L expression (125). The work was done on PBMC of breast cancer patients, where also monocytic activity was increased, suggesting that bromelain could stimulate the suppressed immunocytotoxicity of monocytes at least in a proportion of breast cancer patients (only 40% responded to the enzyme). Opposite effects of bromelain were also reported, such as those blocking signal transduction through Raf-1/MEK/ERK-2 signaling pathway in stimulated T helper cells (126). The inhibition was probably unspecific, as it acted on ERK-2 and p21ras of T cells, however proteolytic-dependent, evoked by selective cysteine protease inhibitor in further experiments.

In another tissue cell model (splenocyte cultures), the enzyme could induce TCR-mediated T cell proliferation, but not CD4^+^ or CD8^+^ lymphocytes, with concomitant inhibition of TCR-mediated IL-2 production albeit T cells growth (127). In addition, the work showed that Ag-specific B cell antibody response was expanded in bromelain-treated mice, though IL-2 mRNA expression was simultaneously blocked. The authors remarked as bromelain could concurrently boost and inhibit T cell responses, depending on the immune environment and consequently, the enzyme could be potentially implicated in T cell autoimmunity. Bromelain was also studied in the innate immune system, where it could enhance and sustain the process. In a recent study, murine macrophages (RAW 264.7 cells and primary macrophages) were challenged with the enzyme, that caused IFN-γ-mediated nitric oxide and TNFα production to strengthen human defences (128). In addition, in murine natural killer, bromelain was able to augment IL-2- and IL-12-mediated IFN- γ production, IL-6, and Gm-CSF, while reducing T helper cells activation.

### 4.5 Inflammation

One important property of bromelain is its anti-inflammatory action. Since ancient times, pineapple as the main source of bromelain has been widely applied against inflammation disorders (34). The main applications of bromelain concern the edema processes of an inflammatory nature in the medical and surgical fields. Bromelain administered i.p. and orally at doses of 5-10, mg/kg reduces edema and inflammation in animals due to histamine, formalin, dextran, carrageenan and egg albumin. At the level of inflammatory tissue, bromelain reduces vasodilation, and increased capillary permeability, leukocyte migration and local pain by inhibiting the formation of bradykinin and serotonin. In addition to these, bromelain exhibited anticancer activity and the ability to induce apoptotic, necrotic and autophagic mechanisms.

More recently, the inflamed colon tissue was used as a model to evaluate the bromelain anti-inflammatory role. For example, in spontaneous colitis (in C57BL/6 IL-10-/- mice) the enzyme was able to reduce histological and clinical signs of inflammation (129). The research was expanded with the use of pineapple juice or purified bromelain in the same animal model, showing that both treatments could lessen colon inflammation and neoplastic lesions (130). Previously in colon biopsies from patients affected by ulcerative colitis and Crohn’s disease, bromelain *in vitro* experiments resulted in diminished amounts of IL-2, IL-6, IL-4, G-CSF, Gm-CSF, IFN-γ, CCL4/macrophage inhibitory protein (MIP)-1β, and TNF, implying a role in inflamed colon against pro-inflammatory cytokines and chemokines (131). Moreover, in the murine inflammatory bowel disease model, a reduction in neutrophil migration was demonstrated through the blockade of the CD128 chemokine receptor (132). Reduced cytokines/chemokines expression and alteration of white blood cell trafficking can impact on tumor microenvironment, another possible immune-mediated and anti-cancer effect of bromelain.

In another inflammatory disorder, bromelain has notably been shown to reduce inflammation in the gastro-enteric environment (133). In particular, using 3 different cell lines as digestion simulated models (AGS, Caco-2, and SW1353 cells), the authors demonstrated that a proprietary bromelain extract could decrease IL-8, COX-2, iNOS, and TNF-α without affecting cell viability. In addition, bromelain inhibited the iNOS and COX-2 expression in LPS-stimulated RAW 264.7 cells presumably as a result of inhibiting ERK (extracellular signal-regulated kinase) and p-38 phosphorylation (134).

Zhou and co-authors have reported that purified fruit bromelain could stop epithelial TNF-α receptors in rat colitis models and intestinal cell line IEC-6 and Caco-2 cells (43). The work reported that colitis symptoms were ameliorated, together with reduced macroscopic damage, decreased mucosal inflammation, and tight junction barrier recovery.

Bromelain can accelerate tissue repair processes as a result of the depolymerization of intercellular structures and modification of vascular permeability (135). The biological effects of bromelain have been partially associated with the modulation of the arachidonic acid cascade (124), similar to another study that showed the alteration of arachidonic acid metabolism with anti-inflammatory and antiplatelet effects of bromelain (135). Bromelain therapy resulted in platelet formation reduction with increased resistance to aggregation (136). It experimentally induced inflammatory reactions in the rat with the interference of eicosanoid formation in the arachidonic acid cascade (124) and could modulate the immune system as an anti-inflammatory factor (71).

Habashi and co-authors have also reported that bromelain potentially exerted anti-inflammatory effects by reducing nitric oxide synthesis *in vitro* without cytotoxicity (137). Bromelain downregulated COX-2 and PGE-2 (prostaglandin E2) expression levels in cells (138). Bhui and co-authors explored that bromelain inhibits COX-2 expression by blocking the activation of MAPK-regulated NF-kappa B against skin tumor initiation triggering the mitochondrial death pathway (139). Bromelain was able to inhibit PGE2 production (56.3%) in doses of 40 mg/kg in the rat (140). In addition, after 10 and 20 mg/kg oral application of bromelain in rats, it reduced inflammations mediators, such as PGE2 and substance P (tachykinin family of peptides) production in plasma and peripheral tissues (141).

Anti-inflammatory effects of bromelain were observed at 100 µg/mL in LPS-induced human U937 macrophages by suppressing macrophage inflammatory protein-1 (MIP-1α and MIP-1β), monocyte chemoattractant protein-1 (MCP-1), IL-8, IL-1 β, IL-6 and COX-2 (142). Moreover, bromelain (50-100 µg/ml) reduced TNF-α, IL-1β and IL-6 from LPS-induced peripheral blood mononuclear cells and monocytic leukemia THP-1 cells (143). Similarly, another work suggested that bromelain could regulate inflammatory cytokines and growth factors, including TNF-α, IL-1β, IL-6, and IFNγ (144). Moreover, 2.5 mg/kg intragastrically application of cross-linked bromelain with organic acids and polysaccharides (CL-bromelain) for 7 days considerably showed anti-inflammatory effects *via* reduction of NF-κB activity and COX-2 mRNA expression in rat livers (145). In addition, CL-bromelain was reported for its suppression of ERK, c-Jun N-terminal kinase, and p38 mitogen-activated protein kinase (145). Kalra and others have reported that bromelain regulates p53, NF-kB, and COX-2 expression by targeting the mitogen-activated protein kinase pathway in mouse skin (90). Plasma prekallikrein and kininogen notably decreased fifteen minutes after a single injection of bromelain (10 mg/kg) in rats and gradually recovered over 72 hours (146).

Another hypothetic role of bromelain is in the inflammation caused by advanced glycation end-products (AGEs). It has been shown that the enzyme could increase the soluble form of receptor for advanced glycation end products (sRAGE) in 11 patients affected by chronic kidney disease (CDK). It is known that kidneys are implicated in the removal of sRAGE and circulating AGEs and non-AGE ligands are present in CKD (128). The authors suggested that the rise of sRAGE in CDK patients was associated with an improved detaching of the cell surface RAGE, however, bromelain’s role in CDK or AGE removal is still unclear, in particular for the small number of patients analyzed (147).

## 5 Synergistic anticancer effects of bromelain combined with chemotherapeutics or other bioactive compounds

Bromelain delivery in association with other molecules is often used to obtain a synergistic effect. Although bromelain alone has shown apoptotic effects in cancer cell lines, other studies combining this enzyme with other compounds or extracts have been developed. An example is the combination of this enzyme with peroxidase, indeed their association produced additive effects with an intensification of intracellular ROS level and disruption of mitochondrial membrane potential, leading to acute myeloid cells (K562) growth inhibition. In addition, they participated in the regulation of the expression of Bax, Bcl2, caspase-3, and cytochrome, which are highly related to apoptosis, as well as promoting a positive regulation of p53, a key molecule involved in cell death (91). In another study, the combination of olive leaf extract (*Olea europaea* L.) and bromelain could have promising effects against lung cancer (93). Combining ethanolic olive leaf extract (EOLE) with bromelain increased Nrf2 translocation from the cytoplasm to the nucleus and terminated the translocation of NF-κB from the cytoplasm to the nucleus. Furthermore, the levels of IL-6 and TNF-α, as well as some metalloproteinases, decreased, suggesting that this association could reduce lung carcinogenesis through the regulation of inflammation and oxidative stress (93).

The combination of curcumin, harpagophytum and bromelain has been shown to reduce inflammation and pain in osteoarthritic human synovial cells, through the decrease of prostaglandin E2, Nerve Growth Factor (NGF), IL-6 (148). Similarly to the previous study, the combination treatment of bromelain, trypsin, and rutin resulted in a significant reduction in pain and inflammation in 103 patients with osteoarthritis of the knee (149). This randomized double-blind study demonstrated that the combination regimen was efficacy in 51.4% of the osteoarthritis patients (while for the control group, diclofenac treatment, was 37.2%), thus sufficiently better to induce the authors to suggest this treatment as a new potential tool for osteoarthritis.

Bromelain may increase the cytotoxicity of cisplatin in the treatment of breast cancer as reported in 2 studies with MDA-MB-231 and 4T1 Breast Tumor cell lines (78, 79). Besides the use of bromelain as a single drug, there are studies in which the effect of bromelain has been combined with other molecules, such as N-acetylcysteine. The combination of the two compounds in different gastrointestinal cancer cell lines showed significant inhibition of cell proliferation, with an increase in autophagosomal markers such as LC3-II (81). In another study, malignant peritoneal mesothelioma (MPM) cells were exposed to the combination of bromelain with cisplatin and fluorouracil, showing cytotoxic effects against these cells in the mixture between bromelain and cisplatin (84).

Bromelain and N-acetylcysteine (NAC) constitute two active ingredients capable of inhibiting the growth and proliferation of induced peritoneal tumors in mice as model animals. Intraperitoneal administration of tumor cells in association with bromelain and NAC showed that bromelain/NAC as a single agent or combination treatment on alternate days inhibited tumor growth and the number of peritoneal nodules in a dose-dependent manner (82). This activity can be traced back to the proteolytic activity of bromelain and the mucolytic activity of NAC. It is known that some mucins, highly glycosylated high molecular weight proteins (i.e. MUC2 and MUC5AC), are involved in the pathogenesis of cancer. Some tumors may use mucins during invasion, metastasis and growth in otherwise inhospitable sites (Byrd & Bresalier, 2004). Besides, extracellular mucus presents a clinically relevant barrier to effective therapy in mucinous cancers. Thus, extracellular mucolytic would decrease compressive effects from bulky mucinous tumor burden. Such an effect was observed when a combination of bromelain + NAC was used to lysate extracellular mucus in the experimental animal model. Bromelain is made up of a mixture of hydrolytic enzymes, not only with endopeptidase activity, hydrolyzes esters, amides and glycosidic bonds, while NAC can reduce disulfide bridges. Such a combination impaired the complex lattice framework of mucus thus improving drug delivery and increasing cytotoxic effects (150).

Bromelain-based mucus-disrupting strategies are also gaining much attention as an effective tool in decreasing the mucus barrier. However, this effect of bromelain may be ineffective, if not harmful, in some circumstances. Non-Small Cell Lung Cancer (NSCLC) is any type of epithelial lung cancer other than small cell lung cancer (SCLC). There are different types of NSCLC which represent about 90% of all lung cancer cases. A significant percentage of these NSCLC cases are characterized by the expression of the anaplastic lymphoma kinase (ALK) protein. Inhibition of ALK is a target for slowing proliferation in NSCLC. Commercial bromelain was tested to evaluate efficacy during the administration of certain ALK inhibitor drugs such as alectinib (ALC), ceritinib (CER), and crizotinib (CRZ). Interestingly, bromelain administration in male Wistar rats caused a significant decrease in plasma levels of CER and CRZ along with an increase in the apparent clearance. However, no significant effect was noticed with ALC (151). Thus, the well-known bromelain muco-permeation enhancing effect can sometimes have unwanted effects.

Even the simple administration of bromelain in mice challenged with 4T1 triple-negative breast cancer cells can increase the effectiveness of anticancer drugs with severe side effects. Animals receiving cisplatin and bromelain jointly showed more significant effects in tumor shrinkage than those receiving bromelain and cisplatin separately by modulating the tumor environmental inflammation (78).

Significant benefits could be obtained if the administration of bromelain allowed the total elimination of synthetic drugs with anticancer activity, which often cause severe side effects. An interesting attempt was made against Dalton’s ascites lymphoma tumor model in Swiss albino mice. When bromelain was used in association with peroxidase, the two enzymatic systems were able to counteract some of the typical effects of lymphoma. In fact, during the progression of the tumor the white blood cell count was increased as well as the number of abnormal leukocytes, while the red blood cell count decreased. These negative effects were mitigated following the administration of bromelain and peroxidase, both enzymatic systems found in some pineapple extracts. This may be due to the increase in tumor cell apoptosis (92) resulting from the increase of pro-apoptotic protein factors and the restoration of ROS levels by controlling the activity of antioxidant enzymes.

A similar approach aims to replace drugs with antioxidant substances extracted from plants rich in phenolic compounds. For this purpose, ethanol extract of *Olea europaea* leaves (EOLE) in combination with bromelain was used to evaluate the amelioration of various hallmarks associated with benzo(a)pyrene-induced lung carcinogenesis in male Swiss albino mice (Majumder et al., 2021). The combined treatment of bromelain and EOLE significantly restored body weight and lung weight in treated animals thus suggesting protection due to the inactivation of excess ROS and/or through inhibition of inflammation or deactivating inflammatory markers. The same kind of antioxidant protection can explain the observed general improvement in lung tissue architecture. Hydroxytyrosol and oleuropein are the main components of the polyphenolic fraction of *Olea europaea*, and their antioxidant potential has long been recognized. On the other side, inflammation provides various molecules essential to the formation of a microenvironment suitable for tumor development. Such molecules include growth factors, survival factors, proangiogenic factors and extracellular matrix-modifying enzymes (Ben-Baruch, 2006). Treatment with bromelain-EOLE reduced some crucial proinflammatory cytokines (TNF-α and IL-6), and also interrupted the NF-kB translocation. Altogether, a combination of EOLE and bromelain alleviated the benzo(a)pyrene-induced lung carcinogenesis associated with pulmonary oxidative stress and inflammation (Majumder et al., 2021).

The various strategies for the fight against cancer include also radiotherapy, the effectiveness of which can be limited by the radioresistance of some tumors as well as by the undesirable effects affecting normal tissues. The development of radiosensitizing drugs has therefore found application in the treatment of tumors by radiotherapy. Radiosensitizers are intended to enhance tumor cell killing while having much less effect on normal surrounding tissues. Some drugs target different physiological characteristics of the tumor, particularly hypoxia of the solid tumor tissue which is considered a major cause associated with radioresistance. From this perspective, the hypothesis that bromelain acts as a radiosensitizer and radioprotector becomes suggestive. Mekkawy and coworkers make this hypothesis their own through *in vivo* studies in Ehrlich solid tumor (EST) bearing mice (152). The size and weight of tumors in gamma-irradiated EST-bearing mice treated with bromelain decreased significantly with a significant amelioration in the histopathological examination (Mekkawy et al., 2020). The benefits could be linked to the gene expression of some nuclear factors (poly ADP ribose polymerase-I, NF-κB), and peroxisome proliferator-activated receptor α together with restored liver function. Besides, bromelain radiosensitizing action could be expressed through increasing lipid peroxidation and ROS production in tumor tissue, inhibition of repair of DNA strand breaks and inhibition of proliferation (153).

## 6 Clinical studies

The administration of bromelain in tumor patients has been also tested in a few numbers of clinical studies and could represent an adjuvant treatment in cancer care. The immunotoxicity effect of monocytes and lymphocytes against target cells of mammary carcinoma (K562 and MDA-MB-231) was tested *in vitro* after 10 days of oral bromelain administration in breast cancer patients (daily up to a dose of 7800 mg) as above mentioned (paragraph 4.4) (Eckert et al., 1999). Eckert and coworkers found that the intake of the bromelain increases the activity b-MAk and MAK of patient monocytes about 2-fold; in the responder patients, it was recorded an increase in cytotoxicity of b-MAK and MK cells respectively from 7.8% to 54% and from 16% to 47%. No detectable IL-1β from monocyte was found in patients before, during and after the treatment in contrast with what was recorded in healthy blood donors, and no effects were observed in NK and LAK-cell activity in the patient cohort. Moreover, the oral intake of bromelain reduced the expression of the cell surface markers CD44 but poorly increased the expression of CD11a and CD62L without changing the level of CD16. These effects were observed also *in vitro* with a higher decrease in the expression of CD16 and CD44. On the whole, the bromelain’s capacity to improve the monocytic cytotoxicity of breast tumor patients makes it suitable as alternative support care in combating cancer.

The anti-cancer properties of bromelain, in combination with N-acetylcysteine, were used also to produce a new drug (BromAc^®^) which has been recently proved effective in the recurrent thoracic pseudomyxoma peritonei (PMP) treatment (154). According to the bromelain-based mucus-disrupting activity (see above), the mucolytic properties of BromAc^®^ help the tumor dissolution when it was injected directly into mucinous disease. However, its effectiveness is related to neoplastic characteristics. Tumors with soft or intermediate textures are more susceptible to the drug’s action. In the first two cases reported, effectively, large response differences were reported, possibly due to the tumor consistency (Lam et al., 2021). The safety profile was however good as also the objective response.

In a phase I study, the safety of bromelain treatment was tested on 20 patients affected by inoperable mucinous cancer. Among the patients, 13 had intra-tumoral treatment and 7 had intraperitoneal treatment (Valle et al., 2021). Intra-tumoral doses consisted of 30-45 mg of bromelain and 1.5 g of NAC while intraperitoneal doses of 45-60 mg of bromelain and 1.5-2 g of acetylcysteine, administrated in 5% glucose. The volume of the fluidized tumor removed was measured and collected for laboratory analysis. The side effects of treatment were recorded after 24 h. Adverse effects were observed in 85% of patients. The main side effects were fever (35%), pain in the injection site (30%), rise in C-reactive protein (CRP)(80%) and white blood cells (WBC) (55%). No death or anaphylactic reactions were recorded. Although the study was carried out on a small number of patients, it was nevertheless promising as in 15 patients (75%) a reduction in tumor-related symptoms after BromAc^®^ administration was reported with manageable side effects.

BromAc^®^ safety was investigated also using blood parameters in 25 patients (mean age 64) with inoperable PMP pre and post-treatment administration (Ke et al., 2021). Patients received bromelain mean dosage of 124 mg and 4.9 g of NAC for each period of treatment, which consisted of an average of 3.8 drug administration on different days. It was tested in the high and low-dose subgroups (mean of 64 mg of bromelain + 2.7 g of NAC and 183 mg of bromelain plus 7.2 g of NAC respectively). An increase in CRP values and WBC, neutrophils and monocytes in both high and low-dose subgroups was found, with a concomitant decrease in albumin and lymphocyte levels suggesting an inflammatory reaction. The other blood parameters, including liver enzymes or coagulation parameters, have not undergone any alteration. This aspect is important in the evaluation of BromAc^®^ safety, that have not shown liver or kidney toxicity.

The role of bromelain (in combination with papain, sodium selenite and *Lens culinaris* lectin) has been also tested as a complementary medicine on more than 600 breast cancer patients to reduce the side effects caused by the administration of the adjuvant hormone therapy. Side effects were measured by scoring from 1 (no side-effects/optimal tolerability) to 6 (extreme side-effects/extremely poor tolerability) The main side effects, arthralgia and mucosal dryness, were drastically reduced after 4 weeks (p<0.001) and 8 weeks (p<0.0001) of treatment (155, 156).

On the whole, these results support further studies on the role of bromelain in the treatment of tumors possibly representing a non-invasive treatment for people suffering from cancer. Despite these promising effects, the number of clinical trials is low and limited to early stages. More efforts are thus desirable to validate a promising new strategy for inoperable neoplastic cases.

## 7 Toxicity and safety data

The recent studies underline its safety and easy of administration with sufficient bioavailability and tolerability both in animal and human studies. Indeed toxicity studies showed a lethal dose of LD50 greater than 10 g/kg in mice, without carcinogenic or teratogenic effects at dosages of 1.5 g/kg per day (157, 158). Other recent studies have shown few side effects arising as a result of taking bromelain in both animals and humans, thus bromelain is considered relatively safe. The main side effects are attributable to the gastrointestinal system (stomach pain and diarrhea), although allergic reactions are always possible in predisposed individuals (159). Bromelain is considered to be safe and it is an effective agent in burn debridement. In Europe, bromelain is approved as a non-steroidal anti-inflammatory agent for oral and topical use for surgical wounds, inflammation, and debridement of deep burns (159, 160). A single 4-hour application of the bromelain-based agent completely debrides deep partial-thickness burns in a validated porcine model (161).

## 8 Limitations, challenges and prospects

Numerous studies in the literature were focused on the anti-cancer role of bromelain, highlighting its importance in cell processes such as apoptosis and necrosis (95). Available data on bromelain also suggested that the enzyme possesses anti-inflammatory activity in acute and chronic inflammation which are associated with cancer. Bromelain can modulate the metabolism of arachidonic acid affecting the production or activity of cytokines and growth factors involved in the inflammatory process. There are numerous published data on the potential mechanism of action of bromelain in anti-inflammatory activity. Nonetheless, they are not exhaustive and leave many questions unanswered: additional anti-inflammatory works are necessary for a better understanding of its mechanism of action. Many preclinical pharmacological studies showed that Bromelain affected cell viability of cancer cells, however, the exact mechanisms of action produced by bromelain are rarely explored. Nonetheless, it is reported that bromelain could act as an immunomodulator by improving the weakened immunocytotoxicity. Other recent studies showed that bromelain stimulates autophagy which is involved in cancer progression and drug resistance. The activation of autophagosome and lysosome formation by bromelain induces cancer cell death, a key factor that should be more profitably exploited in future research.

A complex picture of the effects of bromelain on the immune system is also perceived. Indeed, most of the published papers reported an immunomodulatory activity of bromelain, with a different behavior based on a specific tissue or cell type. Preclinical pharmacological experiments represent the vast majority of research on the effects of bromelain on the immune system, from which it appears that in the future it is necessary to test bromelain in larger clinical studies in humans. Moreover, its cytotoxic effects in cancer cells could be triggered by proteolytic activity, or by platelet aggregation-inhibitory activity or anti-inflammatory properties (16). Tumour cells with such compromised processes are potential targets of bromelain. In addition, it is known that cancer cell death can be induced in different ways, such as *via* apoptosis, necrosis, and autophagy.

The lack of wide clinical trials leaves the opportunity to further test bromelain in humans, giving the possibility to clinicians to lay clear indications of its use. Another issue to be expanded in future research will be bromelain association with other compounds, as above mentioned in some successful works (78, 82, 84, 92–94, 162–164). The possibilities are enormous, and should not be limited to chemotherapeutic drugs (78, 84, 162–164), but expanded to other drug categories, such as anti-hypertensives, hypoglycemics, anti-asthmatics, anti-microbials, immunomodulators, etc. Moreover, it should be kept in mind that bromelain is highly attractive for the industry, given its indispensable nature in foods, cosmetics, pharmaceuticals, and textiles.

A key limitation factor for the successful use of bromelain as a potential anticancer agent is its production and method of extraction: the more these are reduced and the more the market expands. Indeed, more economical but equally effective bromelain will be highly competitive with other products or other sources and will facilitate its use among both the population and industry. This could in turn generate a scientific relapse with the initiation and extension of new and old research in this field. It is expected that in the next future bromelain will be deeply studied and explored, especially focusing on its anti-cancer activity, as this kind of disease is considered the second leading cause of death all over the world (165).

New perspectives and strategies have been proposed to increase the anti-cancer effects of Bromelain from the bench to bedside. The most promising approach is to enhance the bioavailability of bioactive compounds using new pharmaceutical nanoformulations (166). Thus and simple bromelain delivery may require high doses of the active principle also due to the characteristics of the extracellular matrix (ECM) which constitutes a barrier that slows down or prevents the spread of drugs in the parenchyma of the tumor. For this reason, other delivery methods have been investigated. NPs-based delivery systems can offer an effective alternative. The proteolytic activity of bromelain can pave the way for these antitumor drug delivery systems by increasing their penetration into the cell, weakening the resistance of ECM proteins. Thus, doxorubicin, a potent anticancer drug, but with heavy side effects, was delivered in association with chemically immobilized bromelain, by lactobionic acid-modified chitosan NPs. *In vivo* drug biodistribution and antitumor activity in ICR, male mice model lead to higher drug concentration in tumor area and superior antitumor effect (Wang et al., 2018).

Pancreatic cancer is one of the most difficult cancers to treat largely because of the inability of anticancer drugs to penetrate the malignant tissue as a result of the dense ECM (167). This can limit the effect of bromelain in degrading the tumor ECM due to the short half-life of bromelain in the blood. An interesting technology has recently been developed capable of increasing blood retention of proteins termed “Self-assembly PEGylation Retaining Activity (SPRA)” (Higashi et al., 2020). This technology based on reversible poly (ethylene glycol) (PEG) modification was applied to stabilization of bromelain *in vivo*, thus enhancing antitumor activities of doxorubicin and doxorubicin encapsulated in PEGylated liposomes (DOXIL) in tumour-bearing mice. The release rate of doxorubicin can be increased by using nanocarriers prepared by crosslinking bromelain with an ortho-ester-based crosslink agent. The release of bromelain in tumour-bearing mice by pH-sensitive NPs increased the hydrolysis of the ECM favoring the penetration of the anticancer drug (164). Bromelain can also be uploaded to obtain poly(lactic-co-glycolic acid) NPs (bromelain-PLGA NPs). Such a delivery system was evaluated for its anti-cancer efficacy in 7,12-dimethylbenz[a]anthracene (DMBA)-induced and 12-O-tetradecanoylphorbol-13-acetate (TPA) promoted 2-stage skin tumorigenesis model in Swiss albino mice. Bromelain-PLGA NPs showed the most significant chemopreventive and chemotherapeutic effects when compared with free bromelain (Bhatnagar et al., 2015). Bromelain-NPs possibly acted by maintaining a balance between the positive and negative regulators of apoptosis and triggering more cells to apoptosis than free bromelain.

## 9 Conclusions

Bromelain, the main medicinal component of pineapple, was discovered in the late 1800s and since that time research continues to increase available data on its potential benefits. Bromelain is an enzyme with numerous pharmacological properties, as it can act on different health disorders, including osteoporosis and osteoarthritis, diarrhea, chronic wounds, surgical debridement, edema, inflammation, and cancer. Nonetheless, clinical uses of bromelain are limited and poorly representative. The anticancer properties of bromelain are extensively documented *in vitro* experiments, but such demonstrations *in vivo* animal models are far less. The methods and techniques of administration/delivery of bromelain vary significantly. Modalities were tested that involved both the administration of bromelain alone, and in association with other molecules, but the most promising use was the formulation of nanoparticles. Novel approaches to cancer chemotherapy are warmly urgent and bromelain could be regarded as an important tool in the cancer fight.

Several clinical properties have been ascribed to bromelain *vide supra* including anticancer activity. Besides, the utility of these proteins also comprises the cosmetic field, food and beverage production and textile industries as a consequence, the industrial demand in the last few years increased. This phenomenon further affected the final price of the product. However, the protein isolation and purification costs are responsible for more than half of the final commercial price.

## Author contributions

All authors made a significant contribution to the work reported, whether that is in the conception, study design, execution, acquisition of data, analysis, and interpretation, or in all these areas - that is, revising or critically reviewing the article; giving final approval of the version to be published; agreeing on the journal to which the article has been submitted; and confirming to be accountable for all aspects of the work. All authors have read and agreed to the published version of the manuscript.
